# Behavioral effects in disorders of consciousness following transcranial direct current stimulation: A systematic review and individual patient data meta-analysis of randomized clinical trials

**DOI:** 10.3389/fneur.2022.940361

**Published:** 2022-09-29

**Authors:** Zeyu Xu, Ruizhe Zheng, Tiantong Xia, Zengxin Qi, Di Zang, Zhe Wang, Xuehai Wu

**Affiliations:** ^1^Department of Neurosurgery, Shanghai Medical College, Huashan Hospital, Fudan University, Shanghai, China; ^2^National Center for Neurological Disorders, Shanghai, China; ^3^Neurosurgical Institute of Fudan University, Shanghai, China; ^4^Shanghai Clinical Medical Center of Neurosurgery, Shanghai, China; ^5^Bloomberg School of Public Health, Johns Hopkins University, Baltimore, MD, United States

**Keywords:** Disorders of Consciousness (DoC), persistent vegetative state (PVS), minimally conscious state (MCS), transcranial direct current stimulation (tDCS), individual patient data (IPD), meta-analysis

## Abstract

**Background:**

In patients with Disorders of Consciousness (DoC), recent evidence suggests that transcranial direct current stimulation (tDCS) can be a promising intervention for them. However, there has been little agreement on the treatment effect and the optimal treatment strategy for the tDCS in patients with DoC.

**Objective:**

In this meta-analysis of individual patient data (IPD), we assess whether tDCS could improve DoC patients' behavioral performance. We also determine whether these treatment effects could be modified by patient characteristics or tDCS protocol.

**Methods:**

We searched PubMed, Embase, and the Cochrane Central Register of Controlled Trials until 7 April 2022 using the terms “persistent vegetative state,” “minimally conscious state,” “disorder of consciousness,” or “unresponsive wakefulness syndrome,” and “transcranial direct current stimulation” to identify Randomized Controlled Trials (RCTs) in English-language publications. Studies were eligible for inclusion if they reported pre- and post-tDCS Coma Recovery Scale-Revised (CRS-R) scores. From the included studies, patients who had incomplete data were excluded. We performed a meta-analysis to assess the treatment effect of the tDCS compared with sham control. Additionally, various subgroup analyses were performed to determine whether specific patient characteristics could modify the treatment effect and to find out the optimal tDCS protocol.

**Results:**

We identified 145 papers, but eventually eight trials (including 181 patients) were included in the analysis, and one individual data were excluded because of incomplete data. Our meta-analysis demonstrated a mean difference change in the CRS-R score of 0.89 (95% CI, 0.17–1.61) between tDCS and sham-control, favoring tDCS. The subgroup analysis showed that patients who were male or with a minimally conscious state (MCS) diagnosis were associated with a greater improvement in CRS-R score. We also found that patients who underwent five or more sessions of tDCS protocol had a better treatment effect than just one session.

**Conclusion:**

The result shows that tDCS can improve the behavioral performance of DoC patients. The heterogeneity of the treatment effect existed within the patients' baseline conditions and the stimulation protocol. More explorative studies on the optimal tDCS protocol and the most beneficial patient group based on the mechanism of tDCS are required in the future.

**Systematic review registration:**

https://www.crd.york.ac.uk/prospero/, identifier: CRD42022331241.

## Introduction

Disorders of Consciousness (DoC) is defined as alterations in arousal or awareness, often caused by cardiac arrest, traumatic brain injury (TBI), intracerebral hemorrhage, and so on (1). Recent developments in technology in neurological intensive care and neurosurgery have led to more patients surviving. However, the number of patients who do not regain consciousness after these incidents has also increased and these patients often end up with DoC. In the past several decades, a lot of work has been done regarding the diagnosis and prognosis of DoC, advancing our understanding of the condition. However, what is not well known is the therapeutic intervention for DoC. The 2018 American practice guidelines for DoC patients (2) only recommend amantadine for treatment. Many brain stimulation therapy—such as transcranial direct current stimulation (tDCS), transcranial magnetic stimulation (TMS), and deep brain stimulation—have emerged in recent years, but their effects are quite controversial.

Transcranial direct current stimulation (tDCS) is a non-invasive brain stimulation technique that modulates the activity of targeted brain regions by using a low-intensity direct current (usually 1–2 mA) between two electrodes (an anode and a cathode) placed on the scalp (3). Among recent studies, tDCS had shown promising results in DoC. A meta-analysis of non-invasive stimulation treatment in DoC patients demonstrates that tDCS over the dorsolateral prefrontal cortex (DLPFC) can improve the Coma Recovery Score–Revised (CRS-R) score (4). Nevertheless, five recent randomized clinical trials (RCT) reported no treatment effect between treatment and control (5–9). Debate on the efficacy of tDCS in DoC patients is an ongoing one. A possible reason for the conflicting results might be related to different tDCS protocols, such as session numbers and duration, current intensity, stimulation target, and so on. For example, Zhang et al. (10) showed that tDCS over the DLPFC improved behavioral responsiveness in patients, whereas Martens et al. found no improvement over the primary motor cortex (M1). Another possible reason is that because the DoC patients are heterogenous, the difference in treatment effects between patients might be significant. Patients' baseline characteristics, such as age, gender, time from injury, and diagnosis may play an important role. For example, several studies had revealed a better treatment effect in patients with a baseline diagnosis of minimally conscious state (MCS) than a persistent vegetative state (PVS) (11, 12) diagnosis. However, very few studies have discussed the influence of tDCS protocol on patients' characteristics, and uncertainties remain about the treatment effect of tDCS in DoC.

Therefore, we aimed to perform an individual patient data meta-analysis from randomized clinical trials to assess the treatment effect of tDCS. Another purpose of this study was to assess whether patient characteristics or tDCS protocols could modify the treatment effect. We hope through our analysis we could find the optimal tDCS protocol that could most benefit the patient group.

## Methods

### Search strategy and selection criteria

This systematic review and individual patient data meta-analysis is conducted in accordance with the Preferred Reporting Items for Systematic Reviews and Meta-Analyses of individual participant data (PRISMA-IPD) guidelines (13). We searched PubMed, Embase, and the Cochrane Central Register of Controlled Trials until 7 April 2022 using the terms “persistent vegetative state,” “minimally conscious state,” “disorder of consciousness,” or “unresponsive wakefulness syndrome,” and “transcranial direct current stimulation” to identify randomized controlled trials of studies utilizing tDCS as an intervention for DoC patients. The full search strategy is described in Appendix 1 in the Supplementary material. Studies were included if: (1) Studies recruited patients diagnosed with MCS, PVS, or UWS, (2) used tDCS as an intervention, (3) with a sham stimulation as the control; (4) pre- and post- tDCS Coma Recovery Scale-Revised (CRS-R) scores were used to measure the recovery in DoC patients as the outcomes; (5) randomized controlled trials had either cross-over or parallel design; and (6) the authors provided individual patient data. Studies published as conference abstracts, narrative or systematic reviews, or in books were excluded. Additionally, we excluded papers that were not randomized controlled trials or observational studies. Articles that were not accessible in English were also excluded. Two authors (ZYX and RZZ) independently identified studies meeting the inclusion criteria and excluded unrelated studies. Conflicts were resolved by consensus.

### Data collection and management

The following study-level data were extracted: first author, year of publication, study design, numbers of included patients, tDCS protocol with the number of sessions, current intensity, stimulation duration, stimulation site, and any adverse effects reported. Additionally, the following patient-level data were also extracted: age, gender, diagnosis, etiology of injury, time from injury to tDCS intervention, and CRS-R score at baseline and after the intervention. After individual patient data were collected, variables were transformed, when possible, to create a uniform database.

The risk of bias in each trial was assessed by two authors independently using the Cochrane Risk of Bias Tool (14). Two authors (ZYX and RZZ) checked the IPD for all patients. Suspected duplicated patients were discussed and decided whether they should be excluded or not.

### Outcome

The outcome was the behavioral effects of the tDCS treatment, measured *via* the absolute change in CRS-R score between pre-tDCS baseline score and post-tDCS score at the time point after finishing all sessions of tDCS treatment. The CRS-R score is the most commonly used validated behavioral scale for DoC patients (15) and received strong recommendations from recent guidelines (2, 16). It has six sub-scales measuring auditory, visual, motor, verbal, communication, and arousal functions, respectively. The total CRS-R score ranged from 0 to 23, appearing capable of differentiating patients in an MCS group from those in a VS.(17).

### Data analysis

Statistical analysis for outcome of interest was performed with IPD, according to the intention-to-treatment principle. The analysis involved both one-stage and two-stage methods for the outcome. In the one-stage method, a generalized linear mixed-effects model was conducted to analyze all trials simultaneously. In the two-stage method, we first analyzed each trial separately and then used a random-effects meta-analysis model to account for variability between trials and combine them. Adjusted analyses were performed to account for potential baseline incomparability. Adjustments were planned for the following prespecified covariates: age, sex, baseline CRS-R score, etiology of injury, and time from injury to tDCS intervention. Results were reported as the mean difference in treatment effects between treatment and control with accompanying 95% CIs. Heterogeneity was evaluated by *I*^2^, and between-study variance (τ^2^).

We performed the prespecified analysis in the following subgroup: age (adults aged <65 years vs. older adults aged ≥65 years); gender (male vs. female); diagnosis (MCS vs. PVS); etiology of injury (TBI vs. non-TBI; non-TBI included Intracerebral hemorrhage (ICH), anoxia, and stroke); time from injury to tDCS intervention (<3 months vs. more than 3 months); single-session tDCS stimulation protocol vs. multi-session tDCS stimulation protocol; and tDCS stimulation site (DLPFC vs. non-DLPFC). For all subgroup analyses, the same one-stage method was used for analyzing the outcome. Additionally, treatment-by-subgroup interactions were tested by including multiple interaction terms in respective regression models. Subgroup analyses were again adjusted by prespecified covariates.

Moreover, we planned sensitivity analyses for trials with crossover randomized clinical trial study design, by excluding two trials with parallel randomized clinical trial study design. In another sensitivity analysis, we excluded one study which used 4 mA current intensity protocol. All analyses were done with STATA, version 16.0. Forest plots were created by R, version 4.1.3 “forestplot” package.

## Results

### Study and patient characteristics

**Figure 1:**
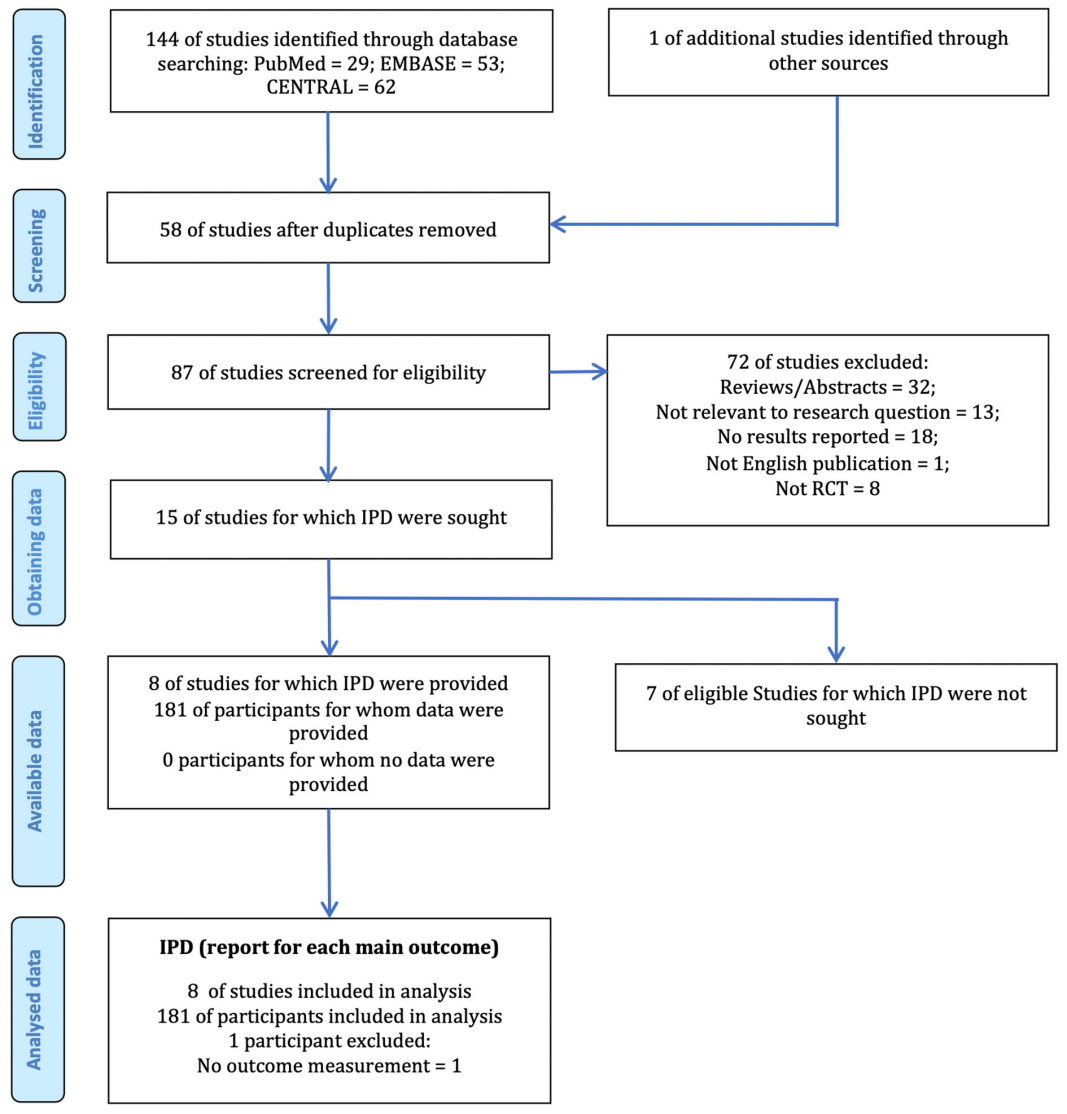
The PRISMA IPD flow diagram. IPD, Individual Patient Data; RCT, Randomized Clinical Trial; PRISMA, Preferred Reporting Items for Systematic Reviews and Meta-Analyses.

Our literature research identified 145 papers from which 58 duplicated studies were removed, and 87 studies were screened by abstract and title. After reviewing the full texts and checking for IPD, six studies that met the eligibility criteria were excluded because no CRS-R score and/or IPD were reported, and finally, eight trials (5–10, 18, 19) were eligible for inclusion in the meta-analysis. Of these, four were done in Belgium, three in China, and one in Italy. The eight trials provided 181 individual patient data, among which one incomplete individual data was excluded from the analysis. We finally included 180 patients in the analyses (Figure 1).

Among the included trials, 41 participants of two trials were in parallel-RCT design, in which 18 participants were allocated to the sham-tDCS group, and 23 participants were allocated to the active-tDCS group; 139 participants from the six trials were in crossover-RCT design, in which all 139 participants were allocated to both active-tDCS group and sham-tDCS group with a washout time interval between the two groups. Because all crossover-RCT designed studies had reasonable washout periods and each crossover trial had pre- and post-tDCS CRS-R scores in both active and sham groups, we included the data from all the allocations for the analyses. A sensitivity analysis was planned to address this. In sum, we had 157 participants who received sham-tDCS and 162 who received active-tDCS.

An overview of the included studies is presented in Table 1. In the pooled study population, the mean (SD) age was 50 (17), 68 (37.8%) patients were female, and 111 (61.7%) were male. The mean (SD) time from injury to tDCS intervention was 29 (56) months; in 79 (43.9%) patients the condition was caused by Traumatic Brain Injury (TBI) and in 101 (56.1%) it was not. In all, 127 (70.6%) patients were diagnosed as MCS at baseline and 52 (28.9%) were diagnosed as PVS. For the stimulation protocol, three studies conducted the single session protocol, two studies had five sessions, two others had ten sessions, and one study had twenty sessions. Of the eight trials, five studies focused on DLPFC as the stimulation site. The duration of the stimulation was 20 min in each trial and all studies used a 2 mA current intensity for stimulation except one which used 4 mA; this study accounted for a sensitivity analysis. Only one study reported a severe adverse effect (epileptic seizure), but that patient quit the trial immediately and was excluded from that study. Thus, all participants in our meta-analysis were well tolerant to the tDCS. Baseline characteristics and protocol characteristics of the 180 patients are shown in Table 2.

**Table 1:** Overview of included studies.

**Reference**	**Design**	**Patients**		**Etiology**		**tDCS protocol**	**Sitimulation target**	**Adverse effect**
Martens et al. (8)	Randomized, sham-controlled crossover study	MCS PVS	29 16	TBI Non-TBI	24 22	1 session, 20 minus 4 mA tDCS 1 session, 20 minus sham tDCS 48 hours washout	Bilateral frontoparietal	None
Wu et al. (9)	Randomized, sham-controlled parallel study	MCS PVS	7 8	TBI Non-TBI	10 5	10 session, 20 minus 2 mA tDCS 10 session, 20 minus sham tDCS	Left/right DLPFC	None
Carriere et al. (5)	Randomized, sham-controlled crossover study	MCS	10	TBI Non-TBI	8 2	1 session, 20 minus 2 mA tDCS 1 session, 20 minus sham tDCS 48 hours washout	Left DLPFC	None
Martens et al. (19)	Randomized, sham-controlled crossover study	MCS PVS	21 6	TBI Non-TBI	15 12	10 session, 20 minus 2 mA tDCS 10 session, 20 minus sham tDCS 8 weeks washout	Left DLPFC	Skin redness in 10 patients, Sleppiness in 3 patients, Epileptic seizure in 1 patients
Martens et al. (7)	Randomized, sham-controlled crossover study	MCS PVS	6 4	TBI Non-TBI	5 5	1 session, 20 minus 2 mA tDCS 1 session, 20 minus sham tDCS 24 hours washout	Left/right M1	None
Zhang et al. (10)	Randomized, sham-controlled parallel study	MCS PVS	15 11	TBI Non-TBI	14 12	20 session, 20 minus 2 mA tDCS 20 session, 20 minus sham tDCS	Left DLPFC	None
Estraneo et al. (6)	Randomized, sham-controlled crossover study	MCS PVS	6 7	TBI Non-TBI	1 12	5 session, 20 minus 2 mA tDCS 5 session, 20 minus sham tDCS 1 week washout	Left DLPFC	None
Huang et al. (18)	Randomized, sham-controlled crossover study	MCS	33	TBI Non-TBI	13 20	5 session, 20 minus 2 mA tDCS 5 session, 20 minus sham tDCS 5 days washout	Posterior parietal cortex	None

Individual patient data were obtained for all trials.

tDCS, transcranial direct current stimulation; MCS, minimally conscious state; PVS, persistent vegetative state; TBI, traumatic brain injury; DLPFC, dorsolateral prefrontal cortex; M1, primary motor cortex.

**Table 2:** Overview of patient and protocol characteristics.

**Characteristics**	**value**
Age, yr	50 ± 17
**Gender**, ***n*** **(%)**
Male	111 (61.7)
Female	68 (37.8)
**Etiology**, ***n*** **(%)**
TBI	79 (43.9)
Non-TBI	101 (56.1)
**Diagnosis**, ***n*** **(%)**
MCS	127 (70.6)
PVS	52 (28.9)
**Time from injury to tDCS**, ***n*** **(%)**
3 and less than 3 months	48 (26.7)
More than 3 months	132 (73.3)
**Numbers of sessions**, ***n*** **(%)**
1 session	66 (36.7)
more than 1 sessions	114 (63.3)
**Stimulation site**, ***n*** **(%)**
DLPFC	91 (50.6)
Non-DLPFC	89 (49.4)

TBI, traumatic brain injury; MCS, minimally conscious state; PVS, persistent vegetative state; DLPFC, dorsolateral prefrontal cortex; tDCS, transcranial direct current stimulation.

The risk of bias was assessed for all trials. Results show the study has a low to moderate risk of bias, which can be seen in Appendix 2.

### Outcome

**Figure 2:**
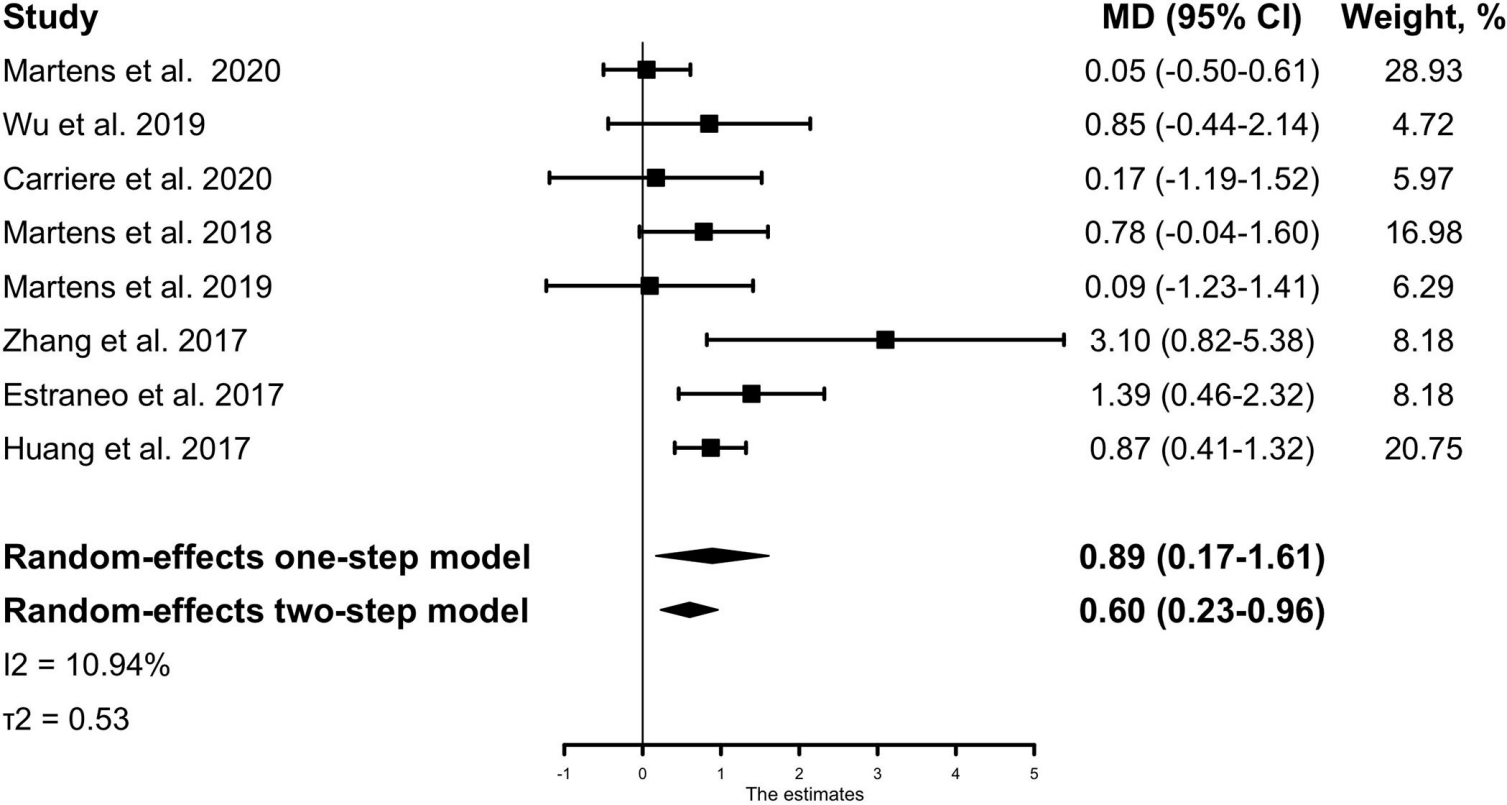
The IPD meta-analysis for all included studies: mean CRS-R score change between pre- and post-tDCS for the treatment group vs. the control group. The Forest plot shows the positive MD both in the one-stage and two-stage meta-analysis method, meaning that active tDCS can significantly improve patients' CRS-R scores than sham tDCS. The mean differences were adjusted for the following variables: age, sex, baseline CRS-R score, etiology of injury, and time from injury to tDCS intervention. MD, mean difference; CRS-R, Coma Recovery Scale-Revised.

The main analysis of the outcome showed a significant result that the treatment effect (mean difference in the absolute CRS-R score change between pre- and post-tDCS for treatment group vs. control group, after adjusting for baseline characteristics) was 0.89 (95% CI, 0.17–1.61, *p* =0.015) with low heterogeneity across studies (*I*^2^ = 10.94%, τ^2^ = 0.53) (Figure 2). It indicates that compared with the control group, tDCS treatment could improve patients' behavior performance measured by the CRS-R score, with a pooled mean difference of 0.89. Similar results could be obtained from the two-stage method, with a mean difference of 0.60 (95% CI, 0.23-0.96, *p* = 0.001). If not stated otherwise, all results are adjusted results.

### Subgroup analysis

**Figure 3:**
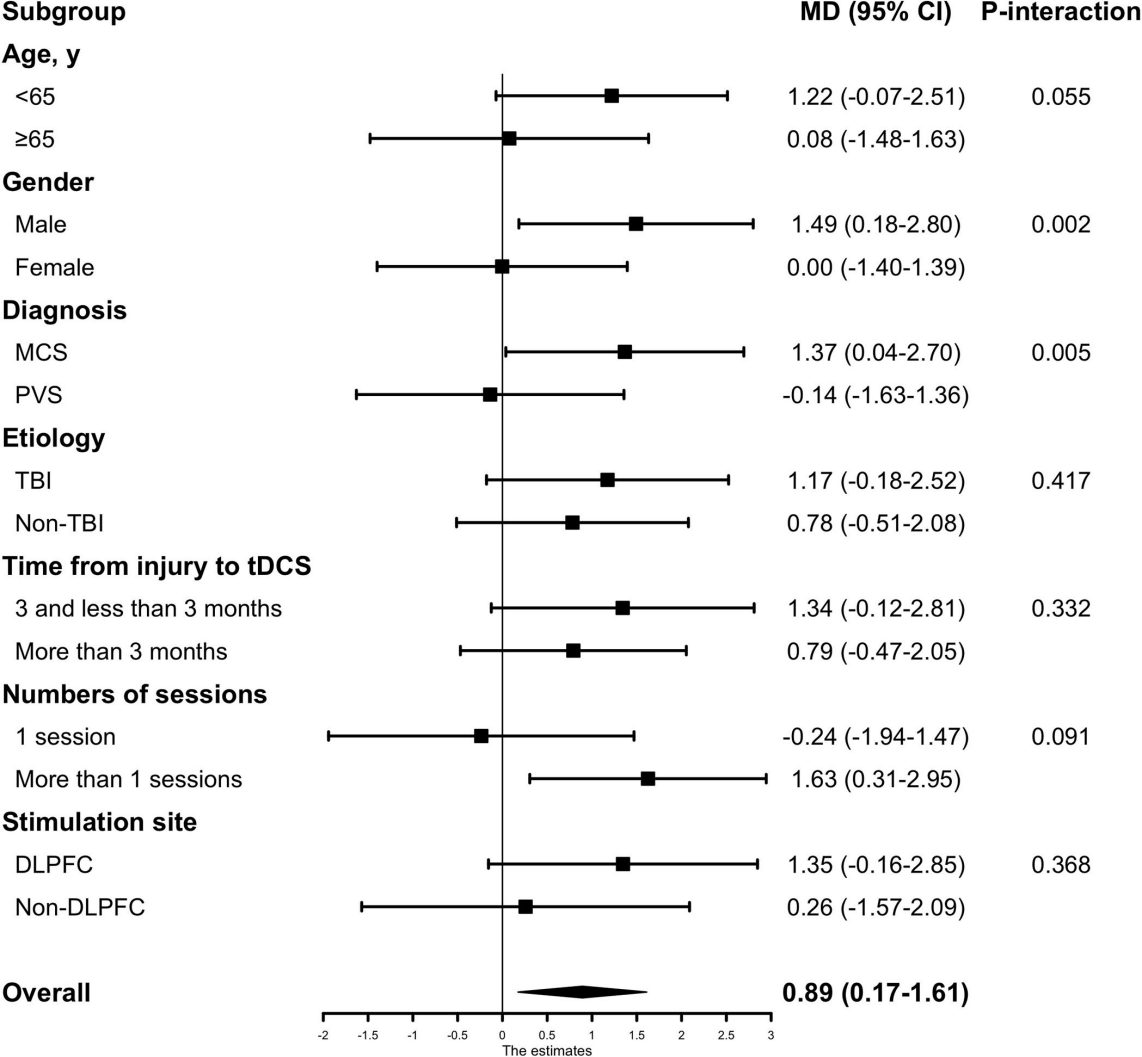
The subgroup analyses based on baseline characteristics and tDCS protocols. Subgroup analyses were adjusted by patients' baseline covariates. Non-TBI included Intracerebral hemorrhage (ICH), anoxia, and stroke. MCS, minimally conscious state; PVS, persistent vegetative state; TBI, traumatic brain injury; DLPFC, the dorsolateral prefrontal cortex.

In the subgroup analysis for the outcome (absolute CRS-R score change after active or sham tDCS), no evidence of heterogeneity of treatment effect was found across any of the following variables: etiology of injury (TBI vs. non-TBI), time from injury to tDCS intervention (<3 months vs. more than three months), or stimulation site (DLPFC vs. non-DLPFC). Among the patient who received only one session, no significant differences in treatment effect were observed between the active and sham groups (−0.24, 95% CI [−1.97–1.47], *p* = 0.786), while in patients who received more than one session, significant improvements in CRS-R score could be observed (1.63, 95% CI [0.31–2.95], *p* = 0.016); but no statistically significant interaction was identified between the session groups and treatment effect (*P*_*interaction*_ = 0.091). Significant heterogeneity of treatment effect was observed for gender where male patients had a mean difference of 1.49 (95% CI, 0.18–2.80) in treatment effect, while female patients had a mean difference of 0.00 (95% CI, −1.40–1.39) in treatment effect (*P*_*interaction*_ = 0.002). Treatment effect in patients' baseline diagnosis also had a significant heterogeneity, where patients diagnosed as MCS had a mean difference of 1.37 (95% CI, 0.04 −2.70) in treatment effect while patients diagnosed as PVS had a mean difference of−0.14 (95% CI, −1.63–1.36) (*P*_*interaction*_ = 0.005) (Figure 3).

A sensitivity analysis excluding two trials that used parallel-RCT design did not show a substantial change in findings that tDCS significantly improved patients' behavioral performance (Appendix 3). Similar results could also be found in another sensitivity analysis in which the only trial that used a 4 mA current intensity protocol was excluded (Appendix 4).

## Discussion

The result of our meta-analysis of individual patient data from eight randomized controlled trials showed that the tDCS treatment effect in patients with DoC was 0.89 (95% CI, 0.17–1.61), providing strong evidence that tDCS treatment is more effective at improving behavioral performance than control. This result was robust in all sensitivity analyses.

In previous research, the behavioral treatment effects in DoC patients following tDCS were controversial. Three observational studies (20–22) and six clinical trials (10, 18, 19, 22–24) were consistent with our findings, whereas seven randomized clinical trials contradicted ours and reported no significant treatment effect on behavior. However, the sample size of these studies was too small, and in most studies the patients' baseline characteristics were unadjusted. It caused a limitation for these studies to properly assess the behavioral treatment effects of tDCS in DoC patients. To our knowledge, the present study is the first meta-analysis that also has individual patient data on tDCS in patients with DoC. In our meta-analysis, individual data of 180 patients from a total of eight studies (5–10, 18, 19) across different countries were used. After adjusting patients' baseline characteristics, our results with a large sample size showed a more credible result than previous studies. Moreover, our meta-analysis of IPD explored the heterogeneity in treatment effect with subgroup analyses.

In our subgroup analysis based on patient characteristics, geriatric patients (age ≥ 65years) and patients under 65 had no heterogeneity of treatment effect (*P*_*interaction*_ = 0.055). This finding should be interpreted with caution because the number of older patients in our study was 36 (20%), so the estimate of treatment effect might be imprecise. Although no significant interaction was detected, there is a trend that patients under 65 showed a more obvious treatment effect than patients over 65 (1.22 vs. 0.08). A larger sample size of older patients could generate more reliable results in future studies. Time from injury to tDCS intervention in patients, whether it was <3 months or more than 3 months, there was no heterogeneity of the treatment effect (*P*_*interaction*_ = 0.332), which indicates that DoC patients in acute-subacute stage and chronic stage would both benefit from tDCS treatment. No significant interaction between the etiology and treatment effect was evident. In the gender subgroup, there was a significant interaction between treatment effect and gender (*P*_*interaction*_ = 0.002). However, previous studies had not reported such a difference before. One reason for this difference might be that no such analysis was undertaken in previous trials because of the relatively small sample size. A significant interaction between the diagnosis subgroup and treatment effect was also observed where the comparison between patients diagnosed with MCS and PVS yielded a *P*_*interaction*_ = 0.005. This result is consistent with two RCTs (11, 12), which reported that the treatment effect was significant in MCS patients but not in PVS patients. In our analysis, the MCS subgroup had an estimated absolute change in CRS-R score of 1.37, much higher than the PVS subgroup of −0.14. This might help guide the clinical utilization of tDCS treatment in DoC patients. Regardless, we believe there might be some tDCS protocols that were effective in PVS patients that need to be researched in the future.

For tDCS protocol, although no statistically significant interaction was identified between session subgroup and treatment effect, the benefit of tDCS in behavioral improvement was significant in patients who underwent multiple sessions of tDCS protocol (1.63, 95% CI [0.31–2.95], *p* = 0.016), but not statistically significant in patients who underwent one session of tDCS stimulation protocol (-0.24, 95% CI [−1.97–1.47], *p* = 0.786). No significant difference was found within the stimulation site subgroups.

In summary, our subgroup analysis suggests that patients who are male or with an MCS diagnosis are most likely to respond to tDCS treatment. In addition, we found that patients under the age of 65 or with multiple sessions of tDCS protocol might benefit from tDCS treatment. Based on our results, we suggest the following tDCS treatment guidance that might help the patients most: firstly, choice of patients—male, under 65 years of age with MCS diagnosis; and secondly, adopt the multi-session tDCS protocol and choose DLPFC as the prior stimulation site. This evidence-based guidance would be more helpful in clinical practice than others. However, it is just a theoretical estimate, and the real effect should be studied further.

### Limitations and future directions

There are some limitations to this study. In most individual studies, they not only assessed the behavioral effect of the tDCS measured *via* CRS-R score but also assessed the neurophysiological effect such as electroencephalogram (EEG) power spectra, EEG complexity, EEG connectivity analyses, and event-related potential (ERP) analyses. Some of them reported that the neurophysiological effect of tDCS could be found even though no behavioral changes were observed (5, 6, 8–11, 25–27). Cavaliere et al. (20) reported that increased connectivity of the extrinsic control network was measured by functional Magnetic Resonance Imaging (fMRI) after tDCS. Thibaut et al. (21) found a transient increase in signs of consciousness after tDCS through fluorodeoxyglucose positron emission tomography (FDG-PET). However, these advanced measurements varied from study to study, making them difficult to be analyzed in the meta-analysis. Hence our study only analyzed the behavioral effect of the tDCS. In future studies, raw data of these neurophysiological assessments might be collected and processed with uniform methods.

Our meta-analysis identified patients and tDCS protocol characteristics associated with an improvement in the CRS-R score. Due to the heterogeneity of included IPD, we had to transform these to create a uniform database to make analysis possible. Thus, some information was lost during the transformation. For example, MCS was sub-stratified into MCS without language (MCS–) and MCS with language (MCS+) (28), a prognostic value might exist in the classification (29). But in our analysis, patients were just divided into MCS and PVS, so the treatment effect between MCS+ and MCS– could not be analyzed. The same situation could also be seen in the etiology of the injury and stimulation site of the tDCS. Future studies might focus on more detailed characteristics and their association with treatment effects. Finally, the interaction analyses in subgroup analysis may be limited to detecting heterogeneity in treatment outcomes, and the results should be interpreted cautiously. Despite the limitations, our study still provides strong evidence for utilizing tDCS in DoC.

## Conclusion

In this meta-analysis of patients with DoC, tDCS intervention could significantly improve patients' clinical behavioral performance measured by the CRS-R score. We found that patients with certain baseline characteristics have a better improvement, while those with other characteristics do not. Future studies should focus on more specific characteristics of patients who respond to tDCS most and identify the tDCS protocol that would benefit patients most based on the mechanism of tDCS and neurophysiology.

## Data availability statement

The original contributions presented in the study are included in the article/Supplementary material, further inquiries can be directed to the corresponding author.

## Author contributions

ZX: study conception, design, data acquisition, formal analysis, visualization, and writing—original draft. TX: investigation and formal analysis. RZ: data acquisition and writing—review and editing. XW: supervision and writing—review and editing. ZQ, DZ, and ZW: data interpretation and revising the manuscript. All authors contributed to the article and approved the submitted version.

## Funding

XW is supported by the Shanghai Municipal Science and Technology Major Project (No. 2018SHZDZX01).

## Conflict of interest

The authors declare that the research was conducted in the absence of any commercial or financial relationships that could be construed as a potential conflict of interest.

## Publisher's note

All claims expressed in this article are solely those of the authors and do not necessarily represent those of their affiliated organizations, or those of the publisher, the editors and the reviewers. Any product that may be evaluated in this article, or claim that may be made by its manufacturer, is not guaranteed or endorsed by the publisher.
